# Barriers and facilitators of mammography screening among Bahraini women: a cross-sectional study in primary care

**DOI:** 10.1186/s12875-026-03367-6

**Published:** 2026-05-18

**Authors:** Jenan Al-Hashimi, Zainab Ahmadi, Muna A. Almutawa, Noor Hamada, Shamma Alfadhel, Fatema Shareeda, Nayla Aldoseri, Amna Aljunaid, Noora Mukhtar, Muneera Almahmeed, Maha AlTajer, Mohamed Hany Shehata

**Affiliations:** 1 Medical Student at the College of Medicine and Health Sciences, Arabian Gulf University, Manama, Kingdom of Bahrain; 2 Family Medicine Consultant, Primary Health Care, Manama, Kingdom of Bahrain; 3 Family and Community Medicine Department, College of Medicine and Health Sciences, Arabian Gulf University, Manama, Kingdom of Bahrain; 4 Family Medicine Department at the Faculty of Medicine, Capital University, Cairo, Egypt

**Keywords:** Breast cancer, Mammography, Screening, Bahrain, Primary Health Care, Barriers, Facilitators

## Abstract

**Background:**

Breast cancer is the most prevalent malignancy among women in Bahrain, with the highest age-standardized incidence rate in the Gulf Cooperation Council (GCC) region. Despite mammography being the gold standard for early detection, screening uptake remains suboptimal. This study aimed to evaluate knowledge, attitudes, and practices regarding mammography screening among Bahraini women and identify barriers and facilitators to its utilization in primary care.

**Methods:**

A cross-sectional analytical study was conducted among 400 Bahraini women aged ≥ 40 years attending five randomly selected primary healthcare centers across Bahrain. Data were collected via face-to-face interviews using a semi-structured questionnaire covering socio-demographics, medical history, knowledge, attitudes, practices, and barriers. Chi-square tests and binary logistic regression were used to identify predictors of screening uptake.

**Results:**

Participants had a mean age of 54.4 ± 8.8 years. Knowledge was predominantly average (47.3%) to good (34.5%). While 70.3% held a positive attitude toward mammography and 68% had been screened at least once, only 43.8% adhered to recommended periodic intervals. The leading enabler was physician recommendation (66.9%). Key barriers included absence of physical symptoms (41.7%), lack of time (41%), and fear of pain or cancer diagnosis (38.9%). Factors significantly associated with higher uptake included older age (*p* < 0.001), higher knowledge (*p* = 0.003), positive family history (*p* < 0.001), history of benign breast disease (*p* = 0.022), and unemployed or retired status (*p* = 0.001). Binary logistic regression confirmed independent predictors: age > 60 years (AOR = 5.84, 95% CI: 2.84–12.01, *p* < 0.001), family history (AOR = 2.39, 95% CI: 1.36–4.20, *p* = 0.002), benign breast disease (AOR = 2.89, 95% CI: 1.26–6.63, *p* = 0.012), and good knowledge level (AOR = 3.23, 95% CI: 1.49–6.97, *p* = 0.003).

**Conclusions:**

Bahraini women demonstrate fair knowledge and positive attitudes toward mammography, yet a significant gap persists between initial screening and periodic adherence. Primary care interventions must move beyond general awareness to address symptom-driven misconceptions and psychological fears. Strengthening physician-led recommendations and implementing electronic reminder systems for high-risk groups are essential to improving national screening program effectiveness.

**Supplementary Information:**

The online version contains supplementary material available at 10.1186/s12875-026-03367-6.

## Background

Breast cancer is the most prevalent malignancy among women globally, accounting for more than one in ten new cancer diagnoses annually [1]. Over the past decade, worldwide incidence has increased by over 20%, accompanied by a 14% rise in mortality [2]. In 2020 alone, the disease was responsible for 2.3 million cases and 685,000 deaths globally [3]. This burden is particularly acute in Bahrain, where breast cancer is the most common malignancy among women. Notably, Bahrain reports an age-standardized incidence rate (ASIR) of 52.3 per 100,000—the highest among all Gulf Cooperation Council (GCC) states [4].

While therapeutic advancements have improved survival rates, early diagnosis remains the most effective strategy for mortality reduction and improved prognosis. Although screening can be performed via breast self-examination (BSE), clinical examination, or ultrasound [5], mammography is recognized as the gold standard for its efficacy in reducing mortality and treatment-related morbidity [6]. Despite its proven benefits, a significantly low proportion of breast cancer cases in Bahrain are detected through screening, highlighting a critical need to investigate the knowledge levels and barriers associated with this modality [7].

In response to the evidence favoring mammography, the Bahrain Cancer Society, in collaboration with the Ministry of Health, launched the National Campaign for the Detection of Breast Cancer in 2005. For over two decades, these organizations have worked to educate the public on the necessity of early diagnosis. Current national guidelines recommend that women of average risk undergo mammography screening every two years starting at age 40 [8].

However, previous research suggests significant gaps in public awareness. A 2011 study involving 286 women at Bahraini primary healthcare centers revealed low general knowledge, with only 5.6% of participants answering more than half of the knowledge-based questions correctly. While higher education was associated with better awareness, healthcare providers were cited as the least common source of information (29.7%) [9]. By 2018, another study in Bahrain found improved awareness regarding the starting age for screening (82.3%) and the safety of the procedure (91.0%); however, only 13.7% of women correctly identified mammography as the most effective detection tool [10].

Similar trends of inadequate knowledge are observed across the region. In Najran, Saudi Arabia, 90.4% of women lacked knowledge regarding mammograms [11], while nearly half of the participants in a Mashhad, Iran, study demonstrated inadequate awareness [12]. Conversely, a Qatari study reported good knowledge of warning signs (69%), though recognition of specific risk factors like early menarche remained low [13]. In the United Arab Emirates (UAE), specifically Ras Al Khaimah, only 4% of participants knew the correct age to begin screening [14], whereas research in India showed higher knowledge levels regarding both the disease and mammography (62.99% and 78.67%, respectively) [15].

Attitudes and practices also vary significantly. In the Eastern Province of Saudi Arabia, only 12.4% of women had undergone mammography, with 75.2% reporting that their physicians played no role in their education regarding screening [16]. In Jordan, while 76% of women were aware of the disease’s prevalence, education level remained the primary predictor of screening knowledge [17]. Regarding attitudes, Bahraini-specific data is currently lacking, but neighboring regions show mixed results. A 2022 Saudi study found that 16.1% of women held poor attitudes toward screening [18], and only 7.9% of Iranian participants reported positive attitudes [12]. In contrast, attitudes in the UAE were generally positive [19], and while Indian women also displayed positive attitudes, their actual screening uptake remained low [15].

Actual screening practice in the region is consistently insufficient. In Bahrain, it is estimated that only 12.7% of cases are detected via mammography [7]. Uptake rates are similarly low in Qatar (16%) [13] and the UAE, where lifetime mammography rates range from 11.7% [20] to 14.7% [14]. Across various Saudi Arabian cohorts, practice rates fluctuate between 12.4% and 28.3%, though one study among university staff reported a higher uptake of 51.5% [11, 16, 18, 21]. Regional rates are even lower in Jordan (6.7% lifetime) [22] and Iran (5.4% annual uptake) [12]. Even in Singapore, participation in routine screening remains below 40% [23].

The barriers preventing women from seeking screening are multifaceted. In Saudi Arabia, the predominant deterrents are fear of a cancer diagnosis (57.2%) and concerns regarding radiation exposure (57%) [18]. In Qatar, logistical issues such as difficulty in securing appointments were identified [13], while UAE participants cited pain and lack of physician recommendations [14]. Iraqi Kurdish women reported a lack of perceived symptoms and time constraints [24], and Jordanian women highlighted the cost of testing and religious beliefs [25]. Internationally, Swedish women cited structural conditions and time estimation [26], while Malaysian women noted a lack of understanding regarding where to access the exam [27].

Participation in screening is influenced by several clinical and demographic factors. Positive correlations have been established between screening uptake and the level of knowledge regarding risk factors and symptoms [17]. Additionally, women over age 40 or those with poorer health status are more likely to undergo mammography [14]. Iranian research confirmed that screening behaviors correlate significantly with both knowledge and attitude scores [12]. Finally, international data from Singapore indicates that lower income, lack of social proximity to cancer patients, and the perception of mammography as “embarrassing” are significant predictors of non-attendance [23].

Despite the high incidence of breast cancer in Bahrain, there is a lack of contemporary research exploring why screening uptake remains low. This study aims to evaluate the knowledge, attitudes, and practices of mammography screening among Bahraini women aged 40 and above attending primary healthcare centers. Specifically, the objectives are to: (1) assess the level of knowledge regarding breast cancer risk factors and screening guidelines; (2) determine the prevailing attitudes toward mammography; (3) measure the actual uptake and frequency of screening; and (4) identify the specific personal and structural barriers that prevent women from utilizing these services. By identifying these factors, this study seeks to provide evidence-based recommendations for primary care interventions to improve early detection rates in the Kingdom of Bahrain.

## Methods

### Study design and setting

A cross-sectional analytical study was conducted among Bahraini women attending primary healthcare centers across the Kingdom of Bahrain. The Gulf Cooperation Council (GCC) is a political and economic alliance of six Arab states: Bahrain, Kuwait, Oman, Qatar, Saudi Arabia, and the United Arab Emirates. These countries share broadly similar sociocultural characteristics, healthcare systems, and epidemiological profiles, making regional comparisons contextually meaningful. Bahrain’s primary healthcare system is structured across five health regions (governorates) and comprises 27 government-operated centers staffed by family physicians and allied health professionals. These centers serve as the first point of contact for preventive and chronic disease services, including national cancer screening programs, and are accessible free of charge to all Bahraini citizens. To ensure geographic representation, a multistage sampling approach was used. First, one primary healthcare center was randomly selected from each of the five health regions using a simple random selection method. The selected centers were: National Bank of Bahrain HC (Muharraq), Sabah Al Salem HC and Yousef Engineer HC (Capital), Hamed Kanoo HC (Northern), and Zallaq HC (Southern).

### Participants and sampling

The target population consisted of Bahraini women aged 40 years and older. Inclusion criteria were Bahraini nationality, age 40 years or older, and no prior diagnosis of breast cancer. Women with a personal history of breast cancer were excluded, as they would be under active surveillance or treatment rather than primary screening. Additionally, women who had undergone prophylactic bilateral mastectomy were excluded, as this procedure eliminates the anatomical basis for mammographic screening. Women who were unfamiliar with mammography completed the demographic and knowledge sections of the questionnaire but were excluded from the attitude and practice analyses, as those items presupposed basic familiarity with the procedure. The number of women in this category is reported in the Results.

Participants were recruited from the waiting areas of the selected health centers using a convenience sampling technique. The sample size was calculated based on the total population of Bahraini females in this age group (*N* = 87,656). Using a 95% confidence level, a 5% margin of error, and an assumed 50% standard deviation, the minimum required sample size was determined to be 383. To account for potential incomplete responses, the final sample was rounded to 400 participants, distributed proportionally across the five centers based on the population size each center serves.

### Study instrument

Data were collected using a semi-structured, six-part questionnaire administered via face-to-face interviews to ensure clarity and response accuracy. The questionnaire was available in both Arabic and English.

Part 1: Demographics: Included age, occupation, educational level, marital status, and presence of children.

Part 2: Medical History: Assessed family history of breast cancer (including degree of kinship) and personal history of benign breast diseases.

Part 3: Knowledge: Contained 15 items evaluating awareness of breast cancer signs, symptoms, risk factors, and mammography guidelines.

Part 4: Attitude: Assessed using a 7-item Likert scale (from “Strongly Disagree” to “Strongly Agree”) regarding the perceived benefits and safety of mammography.

Part 5: Practice: Evaluated whether participants had ever heard of or undergone a mammogram, including the frequency and motivators for screening.

Part 6: Barriers: A list of potential deterrents related to procedures, cancer-related fears, and logistical issues (e.g., time).

### Validity and reliability

The questionnaire was newly developed by the research team, informed by validated instruments from comparable regional KAP studies. Content validity was established through expert review by three Family Medicine and Public Health faculty members, who assessed item relevance, clarity, and domain coverage prior to piloting. Face validity was confirmed during the pilot phase. A pilot study was conducted with 10 participants to assess the questionnaire’s clarity and reliability. The instrument demonstrated excellent overall internal consistency (Cronbach’s alpha = 0.90 for the full instrument). Domain-level internal consistency was also adequate: Knowledge subscale (α = 0.82), Attitude subscale (α = 0.87), and Barriers subscale (α = 0.79).

### Statistical analysis

Data collected via Google Forms were exported to Microsoft Excel and analyzed using IBM SPSS Statistics version 23.0.

#### Knowledge scoring

Correct answers were assigned 1 point, while “no” or “I don’t know” answers received 0. Scores were categorized as poor (< 8), average (8–11), or good (> 11) based on the mean (10 ± 3).

#### Attitude scoring 

Responses were coded as 1 (disagree), 2 (neutral), or 3 (agree). Participants scoring > 19 (the mean) were classified as having a “good” attitude.

#### Statistical tests

Descriptive statistics (frequencies, percentages, means, and standard deviations) were used for demographic data. The relationships between knowledge, attitude, practices, and socio-demographic variables were analyzed using the Chi-square test. Binary logistic regression analysis was conducted with mammography practice (ever screened vs. never screened) as the dependent variable. All variables reaching significance at p < 0.05 in bivariate analysis were entered simultaneously as covariates to generate adjusted odds ratios (AOR) with 95% confidence intervals, controlling for potential confounders. Model adequacy was assessed using the Hosmer–Lemeshow goodness-of-fit test. A p-value of < 0.05 was considered statistically significant.

## Results

A total of 400 Bahraini women participated in the study, with a mean age of 54.4 ± 8.8 years. The demographic distribution (Table 1) shows that the largest age group was 50–60 years (36.0%). Most participants were married (78.3%), and 45.3% were unemployed. Educational attainment varied, with 29.8% holding a bachelor’s degree or higher. Regarding clinical history, 32.8% of women reported a family history of breast cancer, while 13.8% had a personal history of benign breast disease.

**Table 1:** Socio-demographic characteristics and medical history of participants (*N* = 400)

Variables	*N* (%)
Age group	
Less than 50 years	137 (34.3)
50–60 years	144 (36)
More than 60 years	119 (29.8)
Occupation	
Employed	73 (18.3)
Un-employed	181 (45.3)
Retired	146 (36.5)
Educational level	
Less than high school	61 (15.3)
High school	164 (41)
Diploma	56 (14)
Bachelor’s degree and above	119 (29.8)
Marital status	
Single	28 (7)
Married	313 (78.3)
Widowed	31 (7.8)
Divorced	28 (7)
Presence of children	
Yes	343 (92.2)
No	29 (7.8)
Family history of breast cancer	
Yes	131 (32.8)
No	269 (67.3)
History of benign breast diseases	
Yes	55 (13.8)
No	345 (86.3)

The overall knowledge levels were categorized as average for nearly half the sample (47.3%). While awareness of specific signs like breast lumps was high (73.8%), knowledge regarding hormonal risk factors, such as late-age childbearing, was notably lower (33.0%). Most participants (77.5%) correctly identified mammography as the most effective screening.

Table 2 reveals that Many of them considered screening mammography to be worthwhile, willing to do it if offered to them, and safe (90.5%, 77.5%, and 68.3%, respectively). As for the calculated overall attitude scoring, the majority of the participants (70.3%) revealed good attitude towards mammography screening.

**Table 2:** Attitude of participants toward screening mammography

Statement	Agree *N* (%)	Neutral *N* (%)	Disagree *N* (%)
Opinion on the following statements regarding mammography screening			
1. Screening for breast cancer is worthwhile	362 (90.5)	29 (7.2)	9 (2.3)
2. A mammogram detects cancer early, which makes treatment more effective	355 (88.8)	32 (8)	13 (3.3)
3. A mammogram helps find breast lumps easily	334 (83.5)	50 (12.5)	16 (4)
4. A mammogram helps decrease the number of women who die of breast cancer	318 (79.5)	44 (11)	38 (9.5)
5. I will do mammography when it is offered to me	310 (77.5)	47 (11.8)	43 (10.8)
6. A normal mammogram result is a reassurance that no cancer is present	274 (68.5)	64 (16)	62 (15.5)
7. Mammography is considered safe to perform	273 (68.3)	93 (23.3)	34 (8.5)

**Fig. 1:**
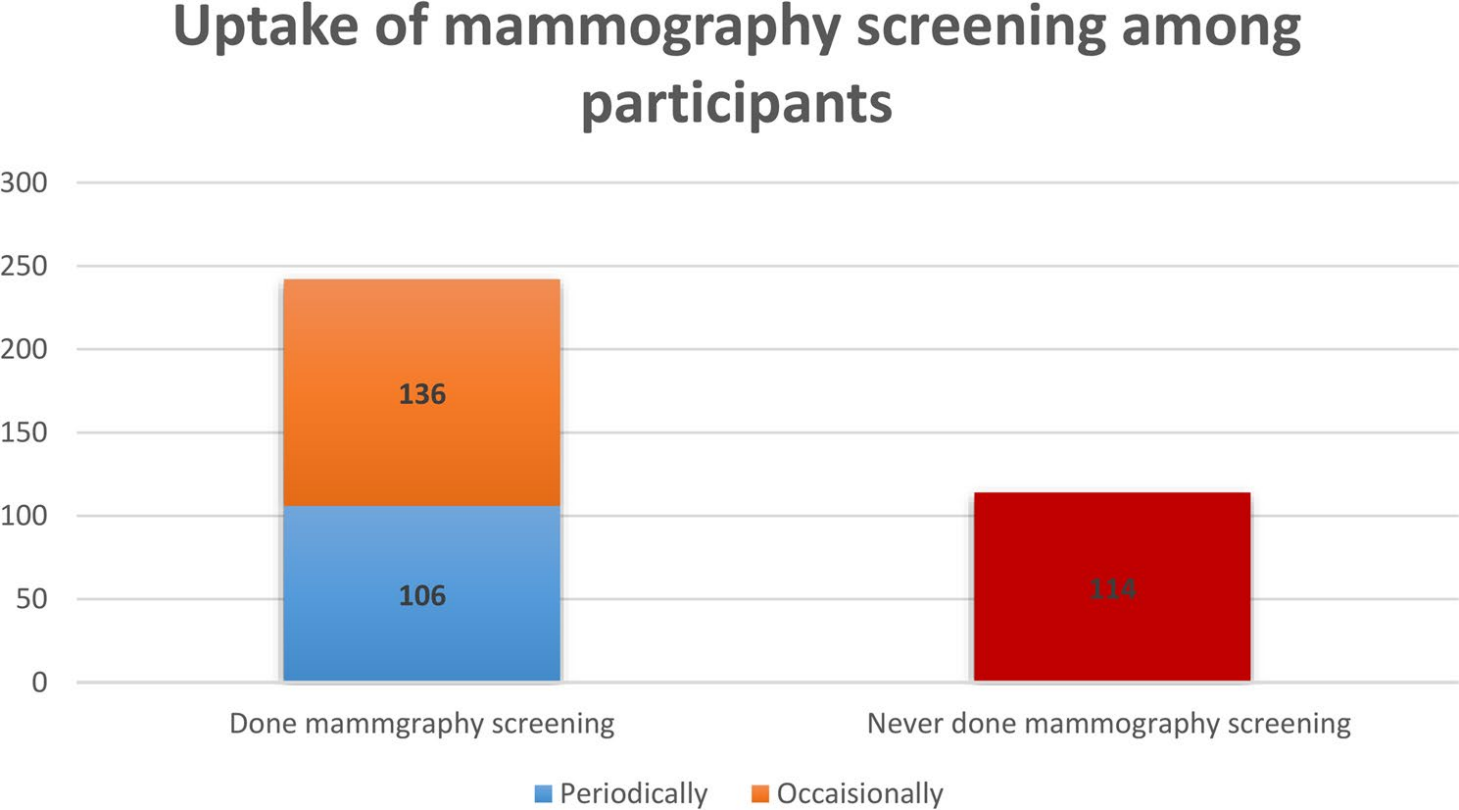
Practicing screening mammogram among participants

Figure 1 demonstrates that a large proportion of the participants had done screening mammography before (68%). Of those who did screening mammography, only 43.8% of them did it periodically as per the guidelines recommendations while 56.2% did it once or twice.

The most frequently cited barriers to mammography included the absence of physical symptoms (41.7%), lack of time (41.0%), and fear of pain or a cancer diagnosis (38.9%) as demonstrated in Table 3.

**Table 3:** Barriers toward screening mammography among participants

Statement	Yes *N* (%)	No *N* (%)
F1: Barriers related to the mammography screening procedure		
1. I am afraid of the pain related to the procedure	56 (38.9)	88 (61.1)
2. I am apprehensive regarding radiation exposure	39 (27.1)	105 (72.9)
3. I am afraid of not knowing the procedure	34 (23.6)	110 (76.4)
4. I feel embarrassed due to a breast-related test	21 (14.6)	123 (85.4)
F2: Barriers related to the thought of not needing the mammogram		
1. I do not have problems with my breast	60 (41.7)	84 (58.3)
2. I do not have a family history of breast cancer	36 (25)	108 (75)
3. I am not old enough to be diagnosed with cancer	10 (6.9)	134 (93.1)
F3: Barriers related to cancer diagnosis		
1. I am afraid of discovering cancer	56 (38.9)	88 (61.1)
2. I believe that cancer has no cure	12 (8.3)	132 (91.7)
F4: Barriers related to time		
1. I do not have time to undergo a screening mammogram	59 (41)	85 (59)
2. I have other priorities to take care of	51 (35.4)	93 (64.6)

**Fig. 2:**
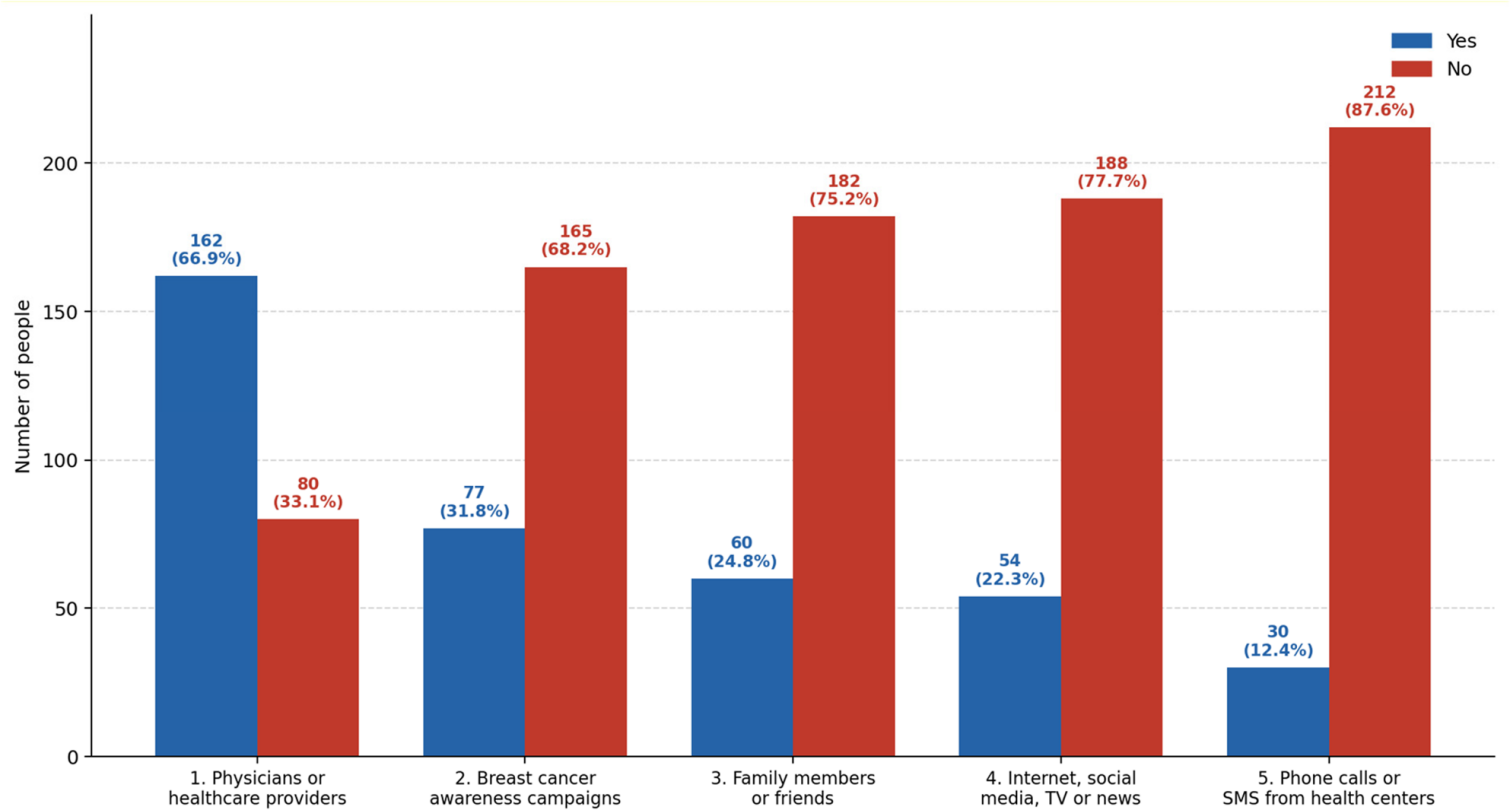
Motivation/reason for doing mammography screening

Based on the data from Fig. 2, the graph illustrates that Physician Recommendation is the most powerful enabler, with 66.9% of women identifying it as their primary motivator. This is followed by Breast Cancer Awareness Campaigns (31.8%) and the influence of Family or Friends (23.4%). Information gathered from the Internet and Social Media (14.6%) and direct outreach via Phone calls or SMS from Health Centers (12.4%) were found to be the least frequent drivers for uptake. These results emphasize the critical role of primary care physicians in directly influencing screening behavior compared to passive media or automated notification systems.

Statistical analysis of the factors associated with mammography uptake (Table 4) revealed several significant clinical and demographic predictors. Age played a critical role (*p* < 0.001), with women over 60 years old being significantly more likely to have undergone screening (85.0%) compared to those under 50 (47.4%). Clinical history was also a powerful driver of behavior, as women with a family history of breast cancer (*p* < 0.001) or a personal history of benign breast disease (*p* = 0.022) demonstrated significantly higher participation rates. Furthermore, knowledge levels were directly linked to practice (*p* = 0.003), with those scoring in the “Good” category showing higher uptake than those with “Poor” knowledge. Occupational status also showed a significant association (*p* = 0.001), likely reflecting differences in time availability between retired and employed women. Conversely, educational level (*p* = 0.580) and marital status (*p* = 0.479) did not significantly influence screening participation, suggesting that in the Bahraini primary care context, clinical risk perception and age-related outreach are more influential than general socio-economic status.

**Table 4:** Factors associated with participation in mammography screening

Factors			Uptake of mammography screening		P value
			Yes N (%)	No N (%)	
Socio-demographic characteristics	Age group	< 50 years	55 (47.4)	61 (52.6)	< 0.001*
		50–60 years	96 (72.2)	37 (27.8)	
		> 60 years	91 (85)	16 (15)	
	Occupation	Employed	33 (49.3)	34 (50.7)	< 0.001*
		Unemployed	105 (69.5)	46 (30.5)	
		Retired	104 (75.4)	34 (24.6)	
	Educational level	< high school	35 (74.5)	12 (25.5)	0.580
		High school	100 (68.5)	46 (31.5)	
		Diploma	35 (70)	15 (30)	
		Bachelor’s	72 (63.7)	41 (36.3)	
	Marital status	Single	15 (57.7)	11 (42.3)	0.479
		Married	188 (67.6)	90 (32.4)	
		Widowed	20 (74.1)	7 (25.9)	
		Divorced	19 (76)	6 (24)	
	Family history of breast cancer	Yes	99 (79.8)	25 (20.2)	< 0.001*
Medical History		No	143 (61.6)	89 (38.4)	
	History of benign breast diseases	Yes	41 (82)	9 (18)	0.022*
		No	201 (65.7)	105 (34.3)	
Level of knowledge		Poor	23 (9.5)	26 (22.8)	0.003*
		Average	121 (50)	49 (43)	
		Good	98 (40.5)	39 (34.2)	
Attitude		Poor	60 (60.6)	39 (39.4)	0.064
		Good	182 (70.8)	75 (29.2)	

The binary logistic regression analysis identified several significant predictors for the uptake of mammography screening among Bahraini women (Table 5). The results revealed that age was a strong predictor: women aged 50–60 years were three times more likely to undergo mammography compared to those under 50 (AOR = 3.006, *p* < 0.001), while women above 60 years were nearly six times more likely (AOR = 5.841; *p* < 0.001). Regarding socio-economic factors, unemployed women were twice as likely to be screened compared to employed women (AOR = 2.060; *p* = 0.042), whereas retired women showed a non-significant trend (AOR = 1.752; *p* = 0.127). A family history of breast cancer significantly increased the likelihood of screening (AOR = 2.390; *p* = 0.002), as did a history of benign breast disease (AOR = 2.886; *p* = 0.012). Finally, knowledge level was a powerful predictor: women with average knowledge were 3.5 times more likely to undergo screening compared to those with poor knowledge (AOR = 3.535; *p* < 0.001), and those with good knowledge were similarly more likely (AOR = 3.227; *p* = 0.003).

**Table 5:** Factors associated with mammography screening uptake among Bahraini women aged 40 years and above: results of binary logistic regression analysis

Factors	Categorical	Adjusted Odds Ratio	95% CI (Lower, Upper)	*P*. value
Age	< 50 years	Ref.		
	50–60 years	3.006	1.665, 5.427	< 0.001
	> 60 years	5.841	2.841, 12.009	< 0.001
Occupation	Employed	Ref.		
	Unemployed	2.060	1.025, 4.138	0.042
	Retired	1.752	0.852, 3.601	0.127
Family history of breast cancer	No	Ref.		
	Yes	2.390	1.360, 4.200	0.002
History of benign breast diseases	No	Ref.		
	Yes	2.886	1.257, 6.626	0.012
Level of knowledge	Poor	Ref.		
	Average	3.535	1.696, 7.368	< 0.001
	Good	3.227	1.494, 6.969	0.003

## Discussion

Breast cancer remains a formidable challenge for women’s health in Bahrain, yet our findings offer a nuanced picture of progress and persistent gaps. The study revealed that most Bahraini women possess a baseline knowledge level ranging from average to good (47.3% and 35.0%, respectively), suggesting that decades of public health messaging have successfully socialized the concept of breast cancer. Like their peers in Qatar, the UAE, Jordan, Iraq, and Hungary, our participants were most adept at recognizing overt symptoms, such as breast lumps or axillary lymphadenopathy [13, 14, 17, 24, 26]. However, this “symptom-based” literacy creates a dangerous paradox: while women know how to spot the disease once it appears, many remain unaware of the silent, hormonal risk factors—such as nulliparity or hormonal replacement therapy—that dictate the need for preventive screening before symptoms arise [13, 17, 24, 28].

When we compare our findings to previous Bahraini data, a shift in risk perception is evident. While Fikree and Hamadeh (2011) and Verhagen et al. (2018) noted family history and smoking as primary concerns, our participants placed even greater emphasis on these factors (88.5% and 71.8%) [9, 10]. This heightened awareness of heredity is particularly vital in the GCC context, where familial ties are strong and genetic risk is high [29].

The psychological landscape of screening in Bahrain appears overwhelmingly positive, with 70.3% of participants maintaining a favorable attitude toward mammography. This mirrors results from Jordan and India but stands in stark contrast to the higher levels of apprehension seen in Saudi Arabia, Iran, and Qatar [11–13, 15, 17].

Furthermore, 77.5% of our sample recognized mammography as the gold standard for early detection, a sharp increase from the 13.7% reported by Verhagen et al. just six years ago [10]. This surge likely reflects the expansion of primary care outreach and the clinical community’s efforts to destigmatize the procedure. However, a “knowledge-practice gap” persists. Although 68% of our participants had undergone a mammogram—a figure significantly higher than that reported among healthcare workers in Saudi Arabia or the general public in the UAE—only 43.8% adhered to the recommended biennial frequency [14, 18, 21]. This suggests that while Bahraini women are willing to initiate screening, they struggle to maintain it as a lifelong habit. The drivers of this behavior have shifted. In 2011,

Bahraini women relied heavily on traditional media (TV and radio) for information [9]. Today, our study identifies Physician Recommendation (66.9%) as the primary motivator, a trend also noted in recent regional studies [21, 29]. This highlights the immense trust Bahraini women place in their primary care providers. Conversely, the most significant barrier was the “illusion of health”—41.7% of women skipped screening simply because they “had no breast problems.” This perceptual barrier, also seen in Singapore and Iraq, underscores a critical misconception: that mammography is a diagnostic tool for the sick rather than a preventive shield for the healthy [23, 24]. Unlike women in Jordan or earlier cohorts in Saudi Arabia, our participants did not cite cost as a barrier, reflecting the success of Bahrain’s free-of-charge screening policy [11, 17].

Statistically, our study clarifies the profile of the “non-screener.” We found that knowledge level is a powerful predictor of practice (*p* = 0.003), as informed women are better equipped to navigate their own health risks [17, 23]. Interestingly, attitude alone did not guarantee action (*p* = 0.064), suggesting that even a woman with a positive outlook may be deterred by logistical or psychological hurdles. Age (*p* < 0.001) and family history (*p* < 0.001) were the strongest predictors of uptake, as older women and those with affected relatives often feel a greater sense of “perceived vulnerability” [14, 21, 23, 29]. Perhaps the most surprising finding was that unemployed and retired women were more likely to be screened than their employed counterparts (*p* = 0.001). This contradicts Jordanian data where employment favored screening [17]. In Bahrain, this suggests that time—or rather, the lack of it—is a structural barrier. When screening services are primarily offered during morning working hours, employed women face a “time tax” that retirees do not. Finally, the lack of association between education level and screening practice (*p* = 0.580) is a departure from UAE and Hungarian findings [14, 28]. It suggests that in Bahrain, the primary care system has effectively leveled the playing field, making screening accessible regardless of a woman’s academic background, provided she receives the right recommendation from her doctor.

Multivariate regression analysis further refines this picture. After adjusting for confounders, age remained the most powerful demographic predictor, with women above 60 years nearly six times more likely to be screened than those under 50 (AOR = 5.84), a magnitude consistent with findings from Malaysia and Oman [30, 31]. The independent contribution of family history (AOR = 2.39) aligns with evidence from Jordan and the broader literature establishing hereditary risk perception as a durable motivator across cultural contexts [31, 32]. Notably, a history of benign breast disease independently predicted uptake (AOR = 2.89), reflecting what Castells et al. termed a “disease experience” pathway to screening — prior contact with the healthcare system for breast-related concerns elevates perceived susceptibility and facilitates adherence [33]. The role of knowledge remained significant in the adjusted model (average-knowledge AOR = 3.54; good-knowledge AOR = 3.23), corroborating Egyptian and Arab immigrant data linking mammography-specific knowledge — not merely general health literacy — to screening behavior [30, 34]. Occupational status lost independent significance for retired women (AOR = 1.75, *p* = 0.127), while unemployed women retained a twofold odds advantage (AOR = 2.06, *p* = 0.042), suggesting that time availability, not retirement status per se, is the operative structural factor.

Implications for Primary Care Practice. The findings of this study carry direct and significant implications for primary care in Bahrain and the broader GCC region. The family physician emerges as the single most influential actor in the mammography screening pathway, with 66.9% of women citing physician recommendation as their primary motivator for screening [30, 35]. This finding is not merely a statistic — it reflects the deep trust that Bahraini women place in their primary care providers and the unique gatekeeping role that family physicians occupy in the preventive healthcare system. Yet, despite this influence, a substantial proportion of women who have never been screened have also never received a recommendation to do so. This gap suggests a systemic failure at the level of opportunistic preventive counseling during routine primary care consultations [36]. Potential reasons include high patient flow, a predominantly reactive (symptom-driven) consultation model, and the absence of structured prompts within electronic health records to flag overdue screening [37]. There is also a mismatch in operating hours: since screening services are predominantly offered during morning working hours, employed women — who represent an estimated 18% of this study’s sample — face a structural time barrier that retired and unemployed women do not. These findings together make a compelling case for embedding brief, standardized mammography counseling prompts into primary care workflows, extending screening service hours or implementing mobile outreach, and establishing a proactive recall system that targets women who have exceeded recommended screening intervals [38]. The primary care center is not simply a point of referral — it is the most promising lever for population-level change in mammography uptake in Bahrain.

## Limitations

Several limitations should be acknowledged when interpreting these findings. First, convenience sampling from waiting areas may have introduced selection bias, as women who regularly attend primary care centers may be more health-seeking than the general population, potentially overestimating knowledge and screening uptake. Second, while the intended sample size was achieved, the data collection period did not permit extension to additional centers or seasonal variation in attendance, which may limit representativeness. Third, the barriers domain did not comprehensively capture all potential deterrents; items relating to religious beliefs, screening accessibility, waiting times, and procedural anxiety were not formally included and should be addressed in future studies. Fourth, a subset of participants reported being unfamiliar with mammography and were therefore unable to complete the attitude and practice sections. These women contributed to the knowledge and demographic analyses but were excluded from attitude and practice assessments. Their profile — typically older age and lower educational attainment — suggests their exclusion may underestimate the burden of low awareness in this subgroup; this has implications for data completeness and the validity of attitude and practice estimates. Fifth, findings are limited to Bahraini women attending public primary healthcare centers and cannot be generalized to non-Bahraini residents, private-sector patients, or those who do not regularly access primary care.

## Conclusion and recommendations

Bahraini females had an average to good level of knowledge about breast cancer; however, they were unaware of some key risk factors such as prolonged use of oral contraceptives or hormonal replacement therapy, being pregnant at an advanced age, or not getting pregnant. They were aware of screening mammography, and had a positive attitude toward screening mammography, but they did not practice it on a regular basis, and 20% of the women who did not practice screening mammography regularly had a positive family history of breast cancer. This high-risk group requires additional attention and regular yearly screening workup by keeping an alerting sign in the electronic system to remind health workers to carefully follow their screening schedule, as well as to send messages by phone and track defaulters. In our study, physicians were the primary motivators for screening mammograms, followed by awareness campaigns. We believe that increasing practice can be improved by social media, reminder texts, or phone calls. The most common reasons for not participating in mammography screenings were a lack of breast complaints, a lack of time, a fear of pain, and a fear of discovering cancer. We recommend that the community learn more about risk factors and regular screening, as well as understand that screening is for asymptomatic people who do not have complaints, and that early detection will reduce morbidity and mortality through social media, posters, leaflets, and videos for people who have had breast cancer discovered by screening mammography to raise awareness. Further research is needed to understand the attitudes and practices of healthcare professionals towards patient screening mammography. From a primary care perspective, these findings underscore the central and largely underutilized role of the family physician in driving mammography adherence. The fact that physician recommendation is the strongest facilitator, yet a meaningful proportion of never-screened women have never received one, reveals a gap that is both preventable and actionable. Primary care interventions must move beyond general public awareness campaigns and instead target the clinical encounter itself: equipping family physicians with brief, structured counseling prompts; leveraging electronic health record alerts for overdue screening; extending clinic hours or implementing community-based outreach to reduce the time burden on employed women; and establishing systematic defaulter-tracking for high-risk groups. Addressing the “symptom-driven” barrier — the misconception that screening is only necessary when symptoms are present — should be a core message in all primary care-based health education, as it was the leading deterrent in this study. These recommendations are directly relevant not only to Bahrain but to the wider GCC region, where primary care systems share comparable structural features and where mammography uptake remains consistently suboptimal.

## Supplementary Information

Supplementary Material 1.

## Data Availability

The datasets used and/or analyzed during the current study are available from the corresponding author on reasonable request.

## References

[1] Alkabban FM, Ferguson T. Breast Cancer. In: StatPearls. Treasure Island (FL): StatPearls Publishing; 2022. Available from: https://www.ncbi.nlm.nih.gov/books/NBK482286/.

[2] World Health Organization. Breast cancer. 2024. Available from: https://www.who.int/news-room/fact-sheets/detail/breast-cancer.

[3] World Health Organization. WHO Breast Cancer. 2021. Available from: https://www.who.int/news-room/fact-sheets/detail/breast-cancer.

[4] Gulf Center for Cancer Control and Prevention. Ten-Year Cancer Incidence among Nationals of the GCC States: 1998–2007. Bahrain Ministry of Health; 2011.

[5] Memorial Sloan Kettering Cancer Center, Mammograms. & Other Types of Breast Exams. 2020. Available from: https://www.mskcc.org/cancer-care/types/breast/mammograms-breast-exams.

[6] Reeves RA, Kaufman T. Mammography. In: StatPearls. Treasure Island (FL): StatPearls Publishing; 2024. Available from: https://www.ncbi.nlm.nih.gov/books/NBK559310/.

[7] Hamadeh RR, Abulfatih NM, Fekri MA, Al-Mehza HE. Epidemiology of breast cancer among Bahraini women: data from the Bahrain Cancer Registry. SQU Med J. 2014;14(2):e176–82. PMC3997533 24790739

[8] Bahrain Cancer Society. Home. 2024. Available from: https://www.bahraincancer.com/.

[9] Fikree M, Hamadeh RR. Breast cancer knowledge among Bahraini women attending primary health care centers. Bahrain Med Bull. 2011;33(4):1–5.

[10] Verhagen K, Khalaf Z, Jassim G. Women’s knowledge of breast cancer: a cross-sectional study. J Womens Health Issues Care. 2018;7(1):1000296.

[11] Alshahrani M, Alhammam SYM, Al Munyif HAS, et al. Knowledge, attitudes, and practices of breast cancer screening methods among female patients in primary healthcare centers in Najran, Saudi Arabia. J Cancer Educ. 2019;34(6):1167–72. 30191519 10.1007/s13187-018-1423-8 PMC6882780

[12] Irani M, Nosrati SF, Ghaffari F, Fasanghari M, Mirzaii K. Knowledge, attitude, and practice of women regarding breast cancer screening behaviors in Mashhad, Iran. J Midwifery Reprod Health. 2021;9(2):2734–43.

[13] Hamed E, Alemrayat B, Syed MA, et al. Breast cancer knowledge, attitudes and practices amongst women in Qatar. Int J Environ Res Public Health. 2022;19(7):4065. 35409678 10.3390/ijerph19073995 PMC8997898

[14] Osmani SSN, Albeshan SM, Mackey M, Brennan PC, Hossain SZ. Understanding better the knowledge, beliefs, and attitudes toward breast cancer and breast screening practices among women living in Ras Al Khaimah, United Arab Emirates (UAE). Int Conf Public Health. 2018.

[15] Pal A, Taneja N, Malhotra N, et al. Knowledge, attitude, and practice towards breast cancer and its screening among women in India: a systematic review. J Cancer Res Ther. 2021;17(6):1314–21. 34916359 10.4103/jcrt.JCRT_922_20

[16] Al-Mulhim FA, Bakr R, Almedallah D, et al. Screening mammography and breast self-examination: attitudes and practices of women in the Eastern Province of Saudi Arabia. Saudi J Health Sci. 2018;7(2):89–100.

[17] Al-Mousa DS, Alakhras M, Hossain SZ, et al. Knowledge, attitude and practice around breast cancer and mammography screening among Jordanian women. Breast Cancer (Auckl). 2020;12:231–42. 10.2147/BCTT.S275445 PMC7666976 33204150

[18] Alenezi AM, Thirunavukkarasu A, Wani FA, et al. Female healthcare workers’ knowledge, attitude towards breast cancer, and perceived barriers towards mammogram screening: a multicenter study in North Saudi Arabia. Curr Oncol. 2022;29(6):4300–14. 35735453 10.3390/curroncol29060344 PMC9222040

[19] Abu Awwad D, Hossain SZ, Mackey M, Brennan P, Adam S. Women’s breast cancer knowledge and health communication in the United Arab Emirates. Front Public Health. 2020;8:564073. 10.3390/healthcare8040495 PMC7711768 33218122

[20] Elobaid YE, Aw TC, Grivna M, Nagelkerke N. Breast cancer screening awareness, knowledge, and practice among Arab women in the United Arab Emirates: a cross-sectional survey. PLoS ONE. 2014;9(9):e105719. 10.1371/journal.pone.0105783 PMC4179300 25265385

[21] Alshammari SA, Alhazmi AM, Alenazi HA, Alshammari HS, Alshahrani AM. Mammography uptake among the female staff of King Saud University. J Family Med Prim Care. 2020;9(1):221–8. 32110594 10.4103/jfmpc.jfmpc_706_19 PMC7014892

[22] Al-Tarawneh MR, Ahram M, Othman A, Shahrouri M. Knowledge, attitudes and practices of breast cancer screening among women in Jordan. Health Care Women Int. 2015;36(5):578–92. 24911030 10.1080/07399332.2014.926900

[23] Liow JJK, Lim ZL, Ho PJ, et al. Attitudes and barriers to mammography screening in Singaporean women through the eyes of their adult children: a focus group study. SSM Qual Res Health. 2022;2:100168.

[24] Amin BA, Babakir-Mina M, Mohialdeen FA, Gubari MIM. Knowledge, attitude and practice toward breast cancer among Kurdish women in Sulaimani Governorate/Iraq. Kurdistan J Appl Res. 2017;2(2):20–8.

[25] Abu-Helalah MA, Alshraideh HA, Al-Serhan AAA, Kawaleet M, Nesheiwat AI. Knowledge, barriers and attitudes towards breast cancer mammography screening in Jordan. Asian Pac J Cancer Prev. 2015;16(9):3981–90. 25987073 10.7314/apjcp.2015.16.9.3981

[26] van Zyl MN, Akhavan S, Tillgren P, Asp M. Experiences and perceptions about undergoing mammographic screening: a qualitative study involving women from a county in Sweden. Int J Qual Stud Health Well-being. 2018;13(1):1410418. 10.1080/17482631.2018.1521256 PMC6147113 30215571

[27] Al-Naggar RA, Bobryshev YV. Practice and barriers of mammography among Malaysian women in the general population. Asian Pac J Cancer Prev. 2012;13(8):3595–600. 23098439 10.7314/apjcp.2012.13.8.3595

[28] Kissné DR, Gede N, Szakács Z, Kiss I. Breast cancer screening knowledge among Hungarian women: a cross-sectional study. BMC Womens Health. 2021;21:120. 33588813 10.1186/s12905-021-01204-9 PMC7885515

[29] Alfailakawi O, Alostath H, Aldubian A, et al. Barriers and limitations for undergoing mammography screenings among Kuwaiti women (aged 40–69) attending primary health care centers. BMC Prim Care. 2025;26:295. 10.1186/s12875-025-02971-2. 41023619 10.1186/s12875-025-02971-2 PMC12482398

[30] Abdullah N, Baharudin N, Mohamad M, Mohamed-Yassin MS. Factors Associated with Screening Mammogram Uptake among Women Attending an Urban University Primary Care Clinic in Malaysia. Int J Environ Res Public Health. 2022;19(10):6103. 10.3390/ijerph19106103. 35627637 10.3390/ijerph19106103 PMC9141597

[31] Al-Azri M, Al-Rubaie K, Al-Ghafri S, Al-Hinai M, Murthi Panchatcharam S. Barriers and Attitudes toward Breast Cancer Screening among Omani Women. Asian Pac J Cancer Prev. 2020;21(5):1339–47.10.31557/. 32458642 10.31557/APJCP.2020.21.5.1339 PMC7541873

[32] Abu-Helalah M, Asfour A, Almadani M, Alfayyadh M, Halayqeh S, Al Tamimi M, Al-Hanaktah M, Al-Rawashdeh I. Uptake rate, barriers, and attitudes towards mammography screening for breast cancer in Jordan. Ann Med. 2025;57(1):2514945. 10.1080/07853890.2025.2514945. 40478644 10.1080/07853890.2025.2514945 PMC12147475

[33] Román M, Louro J, Posso M, Vidal C, Bargalló X, Vázquez I, Quintana MJ, Alcántara R, Saladié F, Del Riego J, Peñalva L, Sala M, Castells X, On Behalf Of The Bele And Iris Study Groups. Long-Term Risk of Breast Cancer after Diagnosis of Benign Breast Disease by Screening Mammography. Int J Environ Res Public Health. 2022;19(5):2625. 10.3390/ijerph19052625. 35270331 10.3390/ijerph19052625 PMC8909630

[34] Salama M. Factors Affecting Mammography Screening Utilization among Educated Women in Al Beheira Governorate, Egypt. Indian J Community Med. 2020 Oct-Dec;45(4):522–5. 10.4103/ijcm.IJCM_41_20. 10.4103/ijcm.IJCM_41_20 PMC7877424 33623214

[35] Sohl SJ, Moyer A. Tailored interventions to promote mammography screening: a meta-analytic review. Prev Med. 2007;45(4):252–61. 10.1016/j.ypmed.2007.06.009 . Epub 2007 Jun 23. PMID: 17643481; PMCID: PMC2078327. 17643481 10.1016/j.ypmed.2007.06.009 PMC2078327

[36] Schrager S, Burnside E. Breast Cancer Screening in Primary Care: A Call for Development and Validation of Patient-Oriented Shared Decision-Making Tools. J Womens Health (Larchmt). 2019;28(2):114–6. 10.1089/jwh.2017.6775. 29757073 10.1089/jwh.2017.6775 PMC6390644

[37] Coma E, Medina M, Méndez L, Hermosilla E, Iglesias M, Olmos C, Calero S. Effectiveness of electronic point-of-care reminders versus monthly feedback to improve adherence to 10 clinical recommendations in primary care: a cluster randomized clinical trial. BMC Med Inf Decis Mak. 2019;19(1):245. 10.1186/s12911-019-0976-8. 10.1186/s12911-019-0976-8 PMC6884876 31783854

[38] Baron RC, Melillo S, Rimer BK, Coates RJ, Kerner J, Habarta N, Chattopadhyay S, Sabatino SA, Elder R, Leeks KJ, Task Force on Community Preventive Services. Intervention to increase recommendation and delivery of screening for breast, cervical, and colorectal cancers by healthcare providers a systematic review of provider reminders. Am J Prev Med. 2010;38(1):110–7. 10.1016/j.amepre.2009.09.031. 20117566 10.1016/j.amepre.2009.09.031

