# Supplementary material for: Barriers and facilitators of mammography screening among Bahraini women: a cross-sectional study in primary care
Source: BMC Prim Care. 2026 May 18;27:262. doi: 10.1186/s12875-026-03367-6 (PMC13362033; doi:10.1186/s12875-026-03367-6)
Supplement: Supplementary file 1 — Supplementary Material 1. [file 12875_2026_3367_MOESM1_ESM.docx]

**Appendix**

**Knowledge, Attitude, Practice, and Barriers Regarding Mammography Screening among Bahraini Women Attending Primary Health Care Centers**

We are a group of medical students from the Arabian Gulf University researching the knowledge, attitude, practices, and barriers regarding mammography screening among Bahraini women attending primary health care centers.

The results will be used solely to obtain insights into mammography practice to encourage Bahraini women to utilize mammography screening for early detection and a better prognosis of breast cancer.

The information obtained will remain confidential, and each participant has the right to withdraw from the study at any time.

When filling out the questionnaire, please ensure no question remains unanswered.

By filling out this questionnaire, you agree to participate in this study.

**مستوى المعرفة، والسلوكيات، والممارسات، والعوائق المتعلقة بفحص الثدي بالأشعة (الماموجرام) لدى النساء البحرينيات اللواتي يرتدن المراكز الصحية للرعاية الأولية بمملكة البحرين**

نحن مجموعة من طلاب طب جامعة الخليج العربي، ونود إجراء مسح لمعرفة المعرفة والسلوكيات والممارسات والعوائق المتعلقة بفحص الثدي بالماموجرام بين النساء البحرينيات اللواتي يحضرن مراكز الرعاية الصحية الأولية.


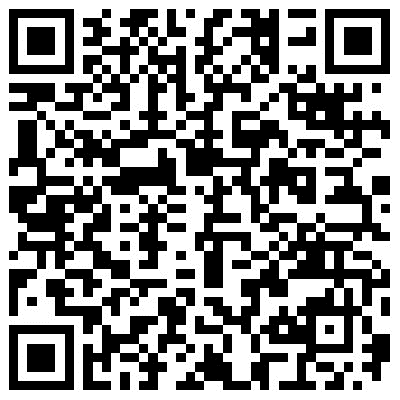
سيتم استخدام النتائج فقط للأغراض العلمية للحصول على رؤى حول ممارسة التصوير الشعاعي للثدي ، بهدف متمثل في تشجيع المرأة البحرينية على استخدام فحص الماموجرام للكشف المبكر والتشخيص الأفضل لسرطان الثدي.

ستبقى المعلومات التي تم الحصول عليها سرية ، ويحق للمشارك الانسحاب من الدراسة في أي وقت.

عند ملء الاستبيان ، يرجى التأكد من عدم ترك أي سؤال دون إجابة.

من خلال ملء هذا الاستبيان، فإنك توافقين على المشاركة في هذه الدراسة.

| **Serial No (case ID):** ………………………… **Local Health Center:** ………………………… **Date of survey:** ………………………… |
| --- |

**Questionnaire**

1. **Demographic characteristic:**

| 1. **Age:** ………………………… (should be from 40 years old and above) |
| --- |
| 1. **Occupation:**  Employed Unemployed Retired |
| 1. **Educational level:** Less than high school High school Diploma Bachelor’s degree and above |
| 1. **Marital status:** Single Married Widowed Divorced |
| 1. **Do you have children?** Yes No (if the answer to Q.4 is single, skip this question) |

**B. Medical history:**

| 1. **Do you have a family history of breast cancer?** Yes No |
| --- |
| **If you answered yes, proceed to the next question. Otherwise, go to question 5.** |
| - **What is your relationship with the relatives in your family who have had a history of breast cancer?**  1. Family history of 1^st^ degree relatives. Yes No 2. Family history of 2^nd^ degree relatives. Yes No 3. Family history of 3^rd^ degree relatives. Yes No 4. Family history of distant relatives. Yes No |
| 1. **Do you have a history of benign breast diseases?** Yes No |

**C. Level of knowledge:**

| Statement | Yes | No | I don’t know |
| --- | --- | --- | --- |
| C1: Do the following statements represent breast cancer signs and symptoms? | | | |
| 1. The presence of a breast lump or enlarged lymph node/s in the armpit |  |  |  |
| 1. Any physical changes in the breast (e.g., itching, skin thickening, changes in the skin texture, redness, and swelling) |  |  |  |
| 1. Changes in the symmetry of the breasts |  |  |  |
| 1. Nipple changes (inversion/retraction) |  |  |  |
| 1. Bloody discharge from the nipple |  |  |  |
| C2: Do the following statements represent breast cancer risk factors? | | | |
| 1. Increasing age |  |  |  |
| 1. Positive family history of breast cancer |  |  |  |
| 1. Having never given birth/pregnancy in old age |  |  |  |
| 1. Smoking and alcohol consumption |  |  |  |
| 1. Using hormonal replacement therapy or oral contraceptive pills |  |  |  |
| C3: Are the following statements about breast cancer mammography screening true? | | | |
| 1. Mammography is the most effective screening tool for the early detection of breast cancer |  |  |  |
| 1. It is preferable to do a mammogram after a clinical examination of the breast |  |  |  |
| 1. Mammography screening starts at age 40 and older |  |  |  |
| 1. Mammography screening should be repeated every 2 years |  |  |  |
| 1. Normal mammogram results still warrant periodic screening |  |  |  |

| **Statement** | **Strongly disagree** | **Disagree** | **Neutral** | **Agree** | **Strongly Agree** |
| --- | --- | --- | --- | --- | --- |
| **What is your opinion on the following statements regarding mammography screening?** | | | | | |
| 1. A mammogram helps find breast lumps easily |  |  |  |  |  |
| 1. A mammogram detects cancer early, which makes treatment more effective |  |  |  |  |  |
| 1. A mammogram helps decrease the number of women who die of breast cancer |  |  |  |  |  |
| 1. Screening for breast cancer is worthwhile |  |  |  |  |  |
| 1. A normal mammogram result is a reassurance that no cancer is present |  |  |  |  |  |
| 1. I will do mammography when it is offered to me |  |  |  |  |  |
| 1. Mammography is considered safe to perform |  |  |  |  |  |

**D. Attitude:**

**E. Practice:**

| 1. **Have you heard of mammography screening before?** Yes No |
| --- |
| **If you answered yes, proceed to question 2. Otherwise, go to section F.** |
| 1. **Have you done mammography screening before?** Yes No |
| **If you answered yes, proceed to question 3. Otherwise, go to section F.** |
| 1. **How often do you do mammography screening?** Periodically (as per the physician orders) Occasionally |
| 1. **How many times have you done mammography screening?** ………………………… times/s |
| - **What/who motivated you to do mammography screening?**  1. Physicians or health care providers Yes No        1. Family members or friends Yes No        1. Breast cancer awareness campaigns or events Yes No        1. Internet, social media, TV or a news article Yes No        1. Phone calls or text messages from health centers Yes No |

**F. Barriers:**

| 1. Do you have any barriers preventing you from undergoing mammography screening? Yes No | | |
| --- | --- | --- |
| If you answered yes, proceed to the next questions. | | |
| Statement | **Yes** | **No** |
| F1: Do you have any of the following barriers related to the mammography screening procedure? | | |
| 1. I am apprehensive regarding radiation exposure |  |  |
| 1. I am afraid of the pain related to the procedure |  |  |
| 1. I am afraid of not knowing the procedure |  |  |
| 1. I feel embarrassed due to a breast-related test |  |  |
| F2: Do you have any of the following barriers related to the thought of not needing the mammogram? | | |
| 1. I’m not old enough to be diagnosed with cancer |  |  |
| 1. I do not have a family history of breast cancer |  |  |
| 1. I don’t have problems with my breast |  |  |
| F3: Do you have any of the following barriers related to cancer diagnosis? | | |
| 1. I am afraid of discovering cancer |  |  |
| 1. I believe that cancer has no cure |  |  |
| F4: Do you have any of the following barriers related to time? | | |
| 1. I have other priorities to take care of |  |  |
| 1. I don’t have time to undergo a screening mammogram |  |  |

**الاستبيان**

| **الرقم التسلسلي:** …………………………………… **المركز الصحي:** ………………………………………… **تاريخ الاستبيان:** ----------------------------- |
| --- |

1. **البيانات الديموغرافية:**

| **١. العمر:** ........................ (من المفترض أن يكون من ٤٠ عاما فما فوق) |
| --- |
| **٢. المهنة :** موظفة عاطلة عن العمل متقاعدة |
| **٣.المستوى التعليمي:** أقل من المرحلة الثانوية المرحلة الثانوية دبلوم شهادة البكالوريس و ما فوق |
| **٤. الحالة الاجتماعية:** عزباء متزوجة أرملة مطلقة |
| **٥. هل لديك أطفال؟** نعم لا (إذا كانت إجابة السؤال ٤ عزباء، الرجاء تخطي هذا السؤال) |

**ب. التاريخ المرضي:**

| **١. هل لديك تاريخ عائلي لمرض سرطان الثدي؟** نعم لا |
| --- |
| **إذا أجبت بنعم، انتقل إلى السؤال التالي. خلافًا لذلك، انتقل إلى السؤال ٥.** |
| - **ما هي علاقتك بأقاربك في عائلتك الذين لديهم تاريخ من الإصابة بسرطان الثدي؟**   **٢.** تاريخ عائلي لأقارب من الدرجة الأولى نعم لا  **٣.** تاريخ عائلي لأقارب من الدرجة الثانية نعم لا  **٤.**  تاريخ عائلي لأقارب من الدرجة الثالثة نعم لا  **٥.** تاريخ عائلي لأقارب بعيدين نعم لا |
| ٦.  **هل لديك تاريخ مرضي من أمراض الثدي الحميدة؟** نعم لا |

**ج. درجة المعرفة:**

| لا أعلم | لا | نعم | العبارة |
| --- | --- | --- | --- |
| ج.١ - هل الأعراض التالية تعتبر من علامات الاصابة بمرض سرطان الثدي؟ | | | |
|  |  |  | ١. وجود كتلة بالثدي أو تضخم في العقد اللمفاوية في الإبط |
|  |  |  | ٢. وجود أي تغيرات في الثدي، مثل (الحكة، سماكة الجلد وتغير نسيجه، احمرار أو تورم في الثدي) |
|  |  |  | ٣. تغيرات في تناسق حجم الثديين |
|  |  |  | ٤. تغييرات في الحلمة (الانقلاب أو الانكماش) |
|  |  |  | ٥. إفرازات دموية من الحلمة |
| ج.٢ - هل العوامل التالية تزيد من نسبة خطر الإصابة بمرض سرطان الثدي؟ | | | |
|  |  |  | ١. التقدم في العمر |
|  |  |  | ٢. وجود تاريخ عائلي لسرطان الثدي |
|  |  |  | ٣. عدم الإنجاب/الإنجاب في سن متأخرة |
|  |  |  | ٤. التدخين وشرب الكحول |
|  |  |  | ٥.استخدام العلاج الهرموني البديل أو حبوب منع الحمل |
| ج.٣ – هل المعلومات التالية المتعلقة بالفحص المبكر لسرطان الثدي بالماموجرام صحيحة؟ | | | |
|  |  |  | ١. فحص الماموجرام هو أكثر أدوات الفحص فعالية للكشف المبكر عن سرطان الثدي |
|  |  |  | ٢.يفضل عمل فحص الماموجرام بعد الفحص السريري للثدي |
|  |  |  | ٣.يبدأ فحص الماموجرام في سن الأربعين فما فوق |
|  |  |  | ٤.يجب تكرار فحص الماموجرام كل عامين |
|  |  |  | ٥. نتائج فحص الماموجرام الطبيعية لا تزال تتطلب الفحص الدوري |

**ه. الممارسات:**

**ه. الممارسات:**

**د. السلوكيات:**

| **أوافق بشدة** | **أوافق** | **محايد** | **لا أوافق** | **لا أوافق بشدة** | **العبارة** |
| --- | --- | --- | --- | --- | --- |
| **ما هو رأيك الشخصي بالعبارات التالية المتعلقة بفحص الماموجرام** | | | | | |
|  |  |  |  |  | ١. يساعد الماموجرام في العثور على كتل الثدي بسهولة |
|  |  |  |  |  | ٢. يكشف الماموجرام السرطان مبكرًا، مما يجعل العلاج أكثر فعالية |
|  |  |  |  |  | ٣. الماموجرام يساعد على تقليل أعداد النساء اللواتي يمتن بسبب سرطان الثدي |
|  |  |  |  |  | ٤. الكشف عن سرطان الثدي بالماموجرام له قيمة وفائدة ويستحق العناء |
|  |  |  |  |  | ٥. تعتبر نتيجة الماموجرام الطبيعية طمأنة بأنه لا يوجد سرطان |
|  |  |  |  |  | ٦. سأقوم بفحص الماموجرام عندما يُعرض علي |
|  |  |  |  |  | ٧. يعتبر فحص الماموجرام آمنًا |

| **١. هل سمعت عن فحص الماموجرام من قبل؟** نعم لا |
| --- |
| **إذا أجبت بنعم، انتقل إلى السؤال التالي. خلافًا لذلك، انتقل إلى الفقرة و من الاسئلة.** |
| **٢. هل قمت بفحص الماموجرام من قبل؟** نعم لا |
| **إذا أجبت بنعم، انتقل إلى السؤال التالي. خلافًا لذلك، انتقل إلى الفقرة و من الاسئلة.** |
| **٣. هل تقومين بفحص الماموجرام بشكل:** دوري ومنتظم عشوائي وغير منتظم |
| **٤. كم مرة قمت بعمل فحص الماموجرام؟** …………………………………… مرة |
| - **ما/من الذي دفعك لإجراء فحص الماموجرام؟**   ٥. الأطباء أو مقدمو الرعاية الصحية نعم لا  ٦. فرد من العائلة أو الأصدقاء نعم لا  ٧. حملات أو فعاليات توعوية بسرطان الثدي نعم لا  ٨. الإنترنت، وسائل التواصل الاجتماعي، التلفزيون، مقال إخباري نعم لا  ٩. المكالمات الهاتفية أو الرسائل النصية من المراكز الصحية نعم لا |

**و. العوائق:**

| ١. هل لديك أي عوائق تمنعك من الخضوع لفحص الماموجرام؟ نعم لا | | | |
| --- | --- | --- | --- |
| إذا أجبت بنعم، فانتقل إلى الأسئلة التالية: | | | |
| لا | **نعم** | **العبارة** | |
| و.١ هل لديك أي من العوائق التالية المتعلقة بإجراء فحص الماموجرام؟ | | | |
|  |  | ١. أنا خائفة من التعرض للإشعاع |  |
|  |  | ٢. أنا خائفة من الألم المرتبط بإجراءات فحص الماموجرام |  |
|  |  | ٣. أنا خائفة لعدم معرفتي بإجراءات الفحص |  |
|  |  | ٤. أشعر بالإحراج من فحص الماموجرام |  |
| و.٢ هل لديك أي من العوائق التالية المتعلقة بفكرة عدم الحاجة إلى فحص الماموجرام؟ | | |  |
|  |  | ١. أنا لست كبيرة بما يكفي ليتم تشخيصي بالإصابة بسرطان الثدي |  |
|  |  | ٢. ليس لدي تاريخ عائلي للإصابة بسرطان الثدي |  |
|  |  | ٣. لا أعاني من مشاكل في الثدي |  |
| و.٣ هل لديك أي من العوائق التالية المتعلقة بتشخيص السرطان؟ | | |  |
|  |  | ١. أخشى اكتشاف إصابتي بسرطان الثدي |  |
|  |  | ٢. أعتقد أن سرطان الثدي ليس له علاج |  |
| و.٤ هل لديك أي من العوائق التالية المتعلقة بالوقت؟ | | |  |
|  |  | ١. لدي أولويات أخرى يجب الاهتمام بها |  |
|  |  | ٢. ليس لدي الوقت الكافي لإجراء فحص الماموجرام |  |
